# Acute aerobic exercise increases exogenously infused bone marrow cell retention in the heart

**DOI:** 10.14814/phy2.12566

**Published:** 2015-10-20

**Authors:** Erica N Chirico, Dennis Ding, Geetha Muthukumaran, Steven R Houser, Tim Starosta, Anbin Mu, Kenneth B Margulies, Joseph R Libonati

**Affiliations:** 1 School of Nursing, University of PennsylvaniaPhiladelphia, Pennsylvania; 2 Temple University School of MedicinePhiladelphia, Pennsylvania; 3 Perelman School of Medicine, University of PennsylvaniaPhiladelphia, Pennsylvania

**Keywords:** Adult stem cells, cell adhesion molecules, myocardial infarction

## Abstract

Stem cell therapy for myocardial infarction (MI) has been shown to improve cardiac function and reduce infarct size. Exercise training, in the form of cardiac rehabilitation, is an essential part of patient care post-MI. Hence, we tested the effects of acute and chronic aerobic exercise on stem cell retention and cardiac remodeling post-MI. Small epicardial MI’s were induced in 12-month-old C57BL/6 mice via cryoinjury. Two weeks post-MI, vehicle infusion (*N* = 4) or GFP^+^ bone marrow-derived cells (BMC) were injected (tail vein I.V.) immediately after acute exercise (*N* = 14) or sedentary conditions (*N* = 14). A subset of mice continued a 5-week intervention of chronic treadmill exercise (10–13 m/min; 45 min/day; 4 days/week; *N* = 7) or remained sedentary (*N* = 6). Exercise tolerance was assessed using a graded exercise test, and cardiac function was assessed with echocardiography. Acute exercise increased GFP^+^ BMC retention in the infarcted zone of the heart by 30% versus sedentary (*P* < 0.05). This was not associated with alterations in myocardial function or gene expression of key cell adhesion molecules. Animals treated with chronic exercise increased exercise capacity (*P* < 0.05) and cardiac mass (*P* < 0.05) without change in left ventricular ejection fraction (LVEF), infarct size, or regional wall thickness (*P* = NS) compared with sedentary. While BMC’s alone did not affect exercise capacity, they increased LVEF (*P* < 0.05) and Ki67^+^ nuclei number in the border zone of the heart (*P* < 0.05), which was potentiated with chronic exercise training (*P* < 0.05). We conclude that acute exercise increases BMC retention in infarcted hearts and chronic training increases exogenous BMC-mediated effects on stimulating the cardiomyocyte cell cycle. These preclinical results suggest that exercise may help to optimize stem cell therapeutics following MI.

## Introduction

Cell-based therapies for the treatment of heart disease, particularly myocardial infarction, hold great promise in regenerating myocardium and reducing the development of heart failure (Segers and Lee 2008; Sanganalmath and Bolli 2013). In both humans and animals, injection or infusion of different types of cardiac and noncardiac-derived progenitor cells have been shown to stimulate new cardiomyocyte formation and improve cardiac function. While the results of these studies have been promising, more work is needed to define the optimal conditions (Oh et al. 2003; Nygren et al. 2004; Chavakis et al. 2008; Mishra 2008). One major problem that has limited the success of cell-based therapy is the low rate of progenitor cell homing and engrafting to the heart, particularly in the face of accelerated apoptosis associated with various types of cardiovascular disease (Barbash et al. 2003; Kwak et al. 2006; Kolwicz et al. 2009). Thus identifying interventions that positively impact the myocardial retention of exogenous progenitors is of great importance.

One potential intervention that might prove useful in optimizing cell therapy is aerobic exercise. Aside from its safety and low cost, aerobic exercise favorably alters the overall metabolic and humoral milieu of the heart and may trigger reparative mechanisms favoring progenitor cell homing and repair (Kolwicz et al. 2009). Our previous work has shown that chronic exercise training increases the abundance of cardiac c-kit^+^ cells and Ki67^+^ cells (Kolwicz et al. 2009), consistent with increased regenerative activity. Moreover, other studies have shown that cardiomyocyte proliferation (Boström et al. 2010) and circulating endothelial progenitors increase with training (Rehman et al. 2004; Brehm et al. 2009). To our knowledge, only one small study has looked at the impact of exercise training on stem cell therapy after MI (Cosmo et al. 2012). Compared to sedentary rats, Cosmo et al. showed an enhanced left ventricular ejection fraction and a more favorable post-MI remodeling profile when low level swimming was coupled to direct mononuclear cell transplantation in the heart (Cosmo et al. 2012). Given that swimming is not often used as an exercise modality in cardiac rehabilitation programs, we designed this study to instead examine the effects of clinically relevant treadmill exercise on the efficacy of stem cell therapeutics. Therefore, the purpose of our experiments was to test the hypothesis that acute aerobic treadmill exercise improves retention of exogenously infused bone marrow-derived cells in the heart and that continued chronic aerobic exercise training would further enhance the efficacy of cell therapy relative to sedentary conditions.

## Materials and Methods

### Experimental paradigm

We performed studies examining how both acute and chronic aerobic training combined with bone marrow cell infusions impacts the heart. In both sets of experiments, cryoinjury-myocardial infarction was induced and a recovery time of 2 weeks was provided. Following recovery from MI, animals assigned to the exercise group ran on a motor driven treadmill for one exercise session. Bone marrow cells were injected immediately post exercise or sedentary conditions. Animals in the acute exercise study underwent echocardiography and were killed 24 h after cell injections. Animals assigned to the chronic exercise training study also had BMC’s injected after their first exercise session, but continued regular treadmill training for 5 weeks before they underwent echocardiography and were killed.

### Experimental myocardial infarction

Female C57BL/6 mice were purchased at 12 months of age (NIH/NIA). To assure a consistent infarct size, myocardial infarction was induced by cryoinjury. Mice were mechanically ventilated under anesthesia with 3% isoflurane and hearts were exposed through a small thoracotomy. A 2 mm diameter metal rod that had been immersed in liquid nitrogen was immediately placed on the anterior wall of the left ventricle for 12–15 sec to induce MI. After allowing a 60-sec re-warming interval, MI was confirmed by a white spot in the region of probe contact and by subsequent histology. Mice were provided a 14 day post-MI recovery period before treadmill exercise and GFP^+^ BMC infusions (i.v., tail vein, 500,000 cells).

### Exercise and bone marrow cell infusion

Mice were randomly assigned into one of three groups: Vehicle Controls (*n* = 4) received neither bone marrow cell (BMC) infusion nor exercise, Sedentary (*N* = 14) animals received BMC infusion but no exercise, and the Exercise group (*N* = 14) received BMC infusions with exercise. Acute exercise consisted of treadmill exercise that gradually increased in 15 min intervals from 8 m/min to 12 m/min, 0 degrees incline, for 45 total minutes. Immediately following acute exercise (within 5 min), GFP^+^ BMC’s (500,000 cells) were intravenously infused into MI animals (tail vein). Twenty-four hours after BMC injections, animals underwent echocardiography and were sacrificed. Hearts were harvested and fixed with 4% paraformaldehyde for histological assessment of GFP^+^ BMC counts, whereas apical tissue sections were flash frozen in liquid N_2_. A subset of mice continued a 5-week intervention of chronic treadmill exercise (10–13 m/min; 45 min/day; 4 days/week; *N* = 7) or remained sedentary (*N* = 6). Forty-eight hours after assessing exercise tolerance with graded exercise tolerance tests as previously described (Sturgeon et al. 2015), cardiac function was measured with echocardiography. Hearts were then harvested and fixed with 4% paraformaldehyde for histological assessment. All procedures conformed to and received approval by the institutional standards of the University of Pennsylvania Animal Care and Use Committee and conformed to American Physiologic Society and NIH standards.

### Echocardiography

Echocardiography was performed by a blinded, experienced sonographer (Small Animal Imaging Facility Core at the University of Pennsylvania) 24 h post BMC infusions for the acute exercise study and 5 weeks post BMC infusions or vehicle infusions in the chronic exercise training study. All mice were sedated using 3% isoflurane and a VisualSonics (Toronto, Canada) 7000 ultrasound machine with a 30 MHz probe was used to guide M-mode acquisition of measurements. A two-dimensional short axis view at the level of the papillary muscles was used to measure left ventricle anterior wall (LVAW) thickness, LV internal diameter (LVID), and LV posterior wall (LVPW) thickness in both diastole (d) and systole (s). Cardiac dimensions were taken using the American Society of Echocardiography leading-edge-to-leading-edge technique. Fractional shortening (FS) was calculated as FS = 100 * [(LVID;d − LVID;s)/LVID;d].

### Bone marrow harvest from GFP^+^ male mice

Homozygous GFP^+^ male animals (C57BL/6 background) served as BMC donors. Following sedation, donor GFP^+^ mice were sacrificed and bone marrow was collected from femurs and tibias by flushing the harvested bone shaft with phosphate-buffered saline (PBS) using a 26 gauge needle. Cells were disaggregated by gentle agitation and RBCs were lysed by incubating for 5 min at room temperature in RBC lysis buffer (eBioscience, San Diego, CA). Following centrifugation, the cells were filtered and suspended in PBS for a final BMC concentration of 5 × 10^5^. The composition of the donor BMC infusate was analyzed in a subset of experiments with flow cytometry gated to CD45^+^ and c-kit^+^ cells. The donor infusate had approximately 5% c-kit^+^ BMC’s and also consisted of over 90% CD45^+^ cells.

### Gene expression

Genomic DNA and RNA or RNA alone were isolated from hearts using the AllPrep DNA/RNA/miRNA Universal kit (Qiagen, Valencia, CA) or Trizol reagent (Invitrogen, Carlsbad, CA), respectively, following manufacturer’s instructions. Genomic DNA and RNA were quantitated spectrophotometrically; integrity of RNA was checked by agarose gel electrophoresis. 200 ng or 1 *μ*g RNA was reverse transcribed to cDNA using the High Capacity cDNA Reverse Transcription Kit (Invitrogen). 1 *μ*L of the reverse transcription reaction or 100 ng of genomic DNA were used for quantitative PCR. GFP and SRY were quantitated using the Taqman probes (Mr04097229_mr and Mm00441712_s1, respectively, Applied Biosystems). The endogenous control used was beta actin (Mm00607939_s1). The mouse Extracellular Matrix and Adhesion molecules array (PAMM 013Z, SABiosciences) was used to profile the expression of cell adhesion and extracellular matrix molecules. The expression of CXCR4 (primer, PPM03149E) was normalized to hsp90 (primer PPM 04803F). All primers were from SABiosciences. Quantitative real-time PCR was performed in an Applied Biosystems 7300 PCR instrument.

### Histology and immunohistochemistry

For fixed tissues, paraffin blocks were cut into 5-*μ*m-thick sections and were mounted on glass slides for staining. Slides were deparaffinized and underwent antigen retrieval in hot citric acid buffer. Stains were conducted against the following proteins: *α*-sarcomeric actin (A2547, Sigma; St. Louis, MO) and EGFP (ab111258, Abcam; Cambridge, MA). The following secondary antibodies were purchased from Jackson Immunoresearch Laboratories (West Grove, PA) and used for detection of primary antibodies as follows: rhodamine red-X donkey anti-Mouse IgM (715-295-020) was used to detect *α*-sarcomeric actin; and FITC donkey anti-goat IgG (705-095-147) was used to detect EGFP. Nuclei in embedded tissues were stained with 4′,6-diamidino-2-phenylindole (DAPI, Millipore; Billerica, MA). Confocal micrographs of all immunostains were acquired using a Nikon Eclipse T1 confocal microscope (Nikon Inc.; Melville, NY). Infarct size was determined with Masson’s trichrome staining. GFP^+^ BMC retention was quantified with fluorescent microscopy assessed from fixed area microscope fields from basal sections of heart and expressed as number of GFP^+^ BMC’s per field. For infarcted animals, Ki67^+^ nuclei and GFP^+^ BMC’s were counted in the infarct zone, the infarct border zone, and in the distal myocardium (1000 *μ*m from the infarct edge).

### Data analysis

Group comparisons for the acute aerobic exercise studies, that is, physical characteristics, histological GFP^+^ BMC cell counts, exercise capacity, echocardiography, and molecular markers, were performed with unpaired *T* tests between groups. Group comparisons for chronic exercise training studies were compared with ANOVA. All data are reported as mean ± SEM and considered significant at an alpha level of 0.05. Statistics were performed with SPSS (Version 20).

## Results

### Acute aerobic exercise

#### One bout of acute aerobic exercise increases BMC retention the post-MI heart

**Figure 1:**
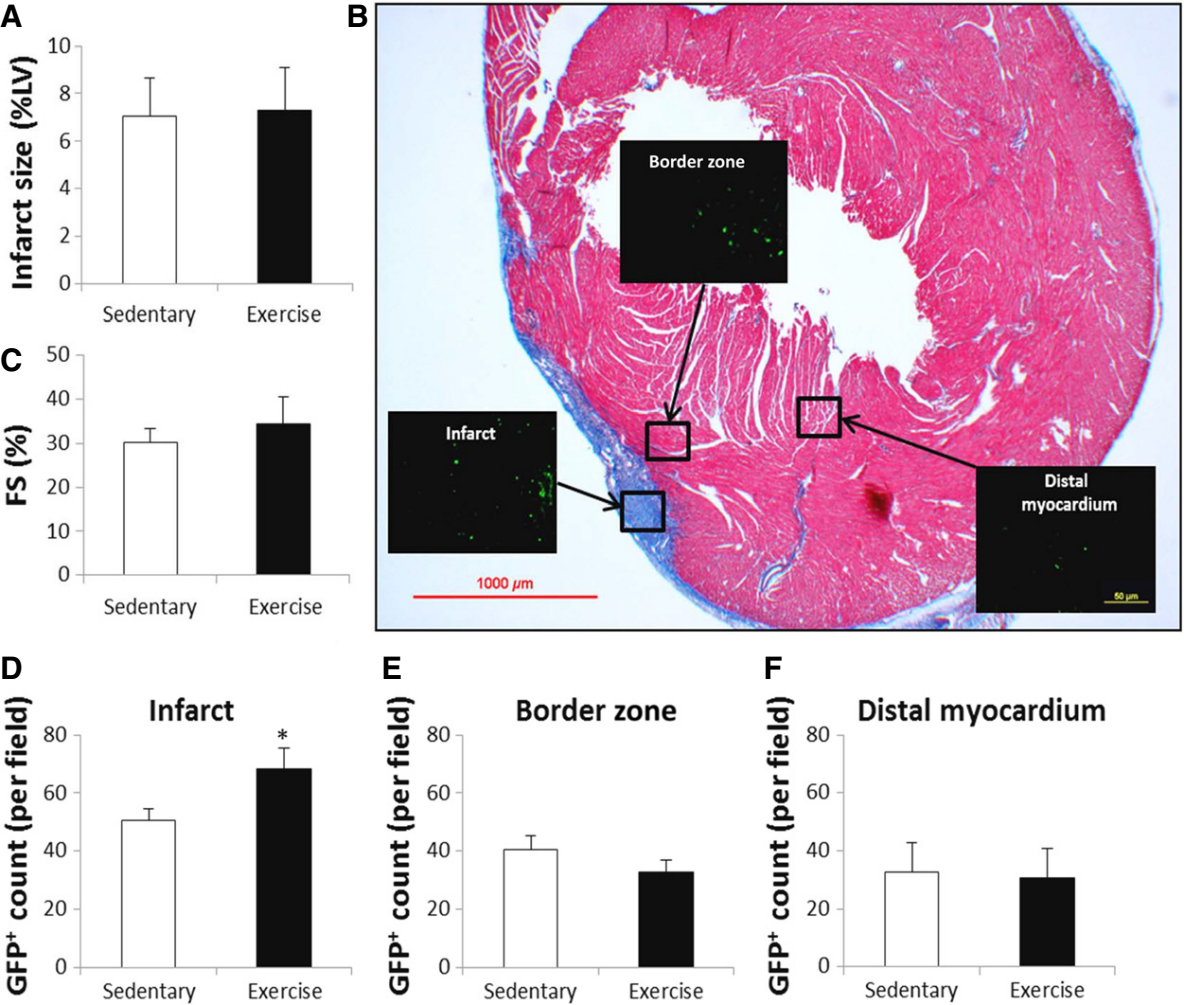
Acute aerobic exercise increases GFP^+^ retention in the heart. Cryoinjury induced small nontransmural MIs (A) that were nearly identical between sedentary and exercise hearts. Panel B shows a representative Massons trichrome stained cross section. Fractional shortening was similar between sedentary and exercise hearts (C). GFP^+^ BMC’s were measured in the infarct, border zone, and distal myocardium (1000 *μ*m from infarct) (Panel B). GFP^+^ BMC retention was greatest in the infarct region and was increased with exercise (D). Less GFP^+^ BMC retention was seen in the border zone and distal myocardium, and was not significantly different between sedentary and exercise hearts (E and F). * indicates *P* < 0.05 versus sedentary.

**Figure 2:**
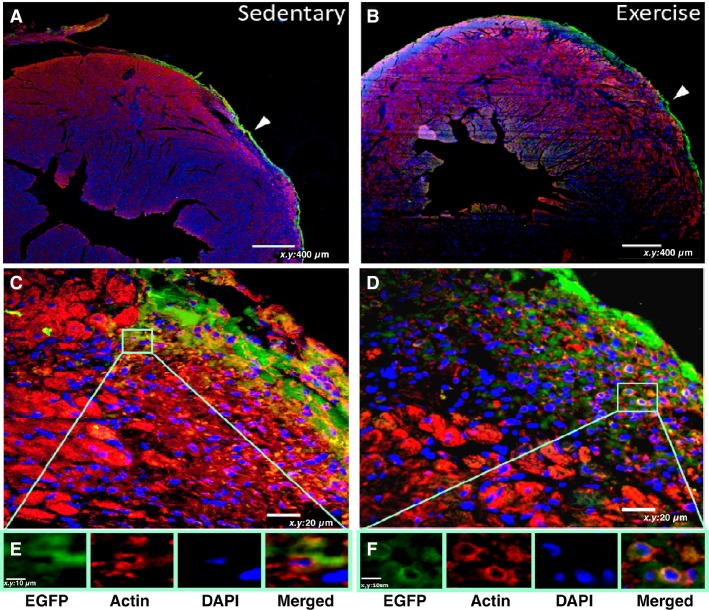
Confocal Imaging of Infarct Region in Sedentary and Acute Exercise. Representative samples of heart sections were immunostained and imaged with confocal microscopy. Injected BMC’s are labeled with green (EGFP), nuclei are labeled with DAPI (blue), and sarcomeric actin (red). Top row: Low magnification view of myocardial infarct (MI) area in sedentary (A) and exercised (B) mice. Middle row: Higher magnification of infarct area in corresponding mice. Bottom row: squared area at higher magnification showing individual and merged staining. Exercised hearts showed more actin-containing nucleated GFP^+^ cells compared to sedentary hearts.

There were no differences in physical characteristics between sedentary and exercise post-MI mice (Table1). Cryoinjury induced a small (7%) nontransmural MI that was similarly sized between sedentary and exercise hearts (Fig.1A and B). Fractional shortening was well maintained post-MI and likewise similar between sedentary and exercise hearts (Fig.1C). There were no differences in regional wall thickness, stroke volume, LV mass or E/A ratio between exercise, and sedentary hearts (Table2). GFP^+^ BMC’s were analyzed in the infarct, border, and distant myocardial zones (Fig.1B, D–F). Exercise increased GFP^+^ BMC retention in the infarct zone by over 30% relative sedentary controls (*P* < 0.05). BMC retention declined in the distant myocardium relative to the infarct area, and exercise did not promote GFP^+^ BMC retention in the either the border or distant myocardial zones. In fact, so few BMC’s were retained in the border or distant myocardium, consistent PCR signals for GFP^+^ and SRY were not detectable in heart extracts. In a subset of the retained GFP^+^ BMC’s in the infarct zone, co-expression with sarcomeric actin was visible (Fig.2). Also, because increased coronary flow may have been a mechanism by which acute aerobic exercise increased BMC retention, we increased cardiac output in sedentary animals with MI using dobutamine (12 ng/mg/min for 6 min), followed by the infusion of 5 × 10^6^ bone marrow cells to mimic an exercise stimulus. The results of these studies showed undetectable levels of BMCs (as indexed by PCR-derived SRY) in the heart. As a positive control we did show consistent SRY signals in the spleen which were not different between vehicle control and dobutamine infusions (Vehicle control: 3.7 ± 0.9 delta CT vs. Dobutamine: 3.6 ± 0.7 delta CT, *P* = NS),

**Table 1:** Baseline physical characteristics

	Sedentary (*n* = 7)	Exercise (*n* = 7)	*P*-value
Body weight (g)	25.9 ± 0.6	25.6 ± 0.8	0.86
Heart weight (mg)	145 ± 7.9	137 ± 7.4	0.45
Heart:Body weight (mg/g)	5.62 ± 0.22	5.36 ± 0.33	0.54
Tibia length (cm)	1.89 ± 0.01	1.89 ± 0.01	0.51
Heart/Tibia (mg/cm)	77 ± 4.0	73 ± 3.9	0.48
Wet:Dry lung weight	4.0 ± 0.1	4.0 ± 0.3	0.89

**Table 2:** Baseline echocardiography

	Sedentary	Exercise	*P*-value
LVID;d (mm)	3.88 ± 0.2	3.47 ± 0.1	0.17
LVPW;d (mm)	0.91 ± 0.01	1.05 ± 0.4	0.45
LVID;s (mm)	2.70 ± 0.2	2.25 ± 0.2	0.16
LVPW;s (mm)	1.26 ± 0.2	1.42 ± 0.1	0.42
LV Vol;d (*μ*L)	66.6 ± 8.9	50.2 ± 4.7	0.15
LV Vol;s (*μ*L)	29.0 ± 6.3	18.8 ± 3.6	0.20
Stroke volume (*μ*L)	31.4 ± 3.6	37.6 ± 4.7	0.16
LV Mass (mg)	122 ± 12	123 ± 15	0.94
E/A	1.85 ± 0.5	1.28 ± 0.1	0.52

LVID, left ventricular internal dimension; LVPW, left ventricular posterior wall; d, diastolic; s, systolic; E/A, early to late ventricular filling velocity.

#### Cell adhesion molecules

**Figure 3:**
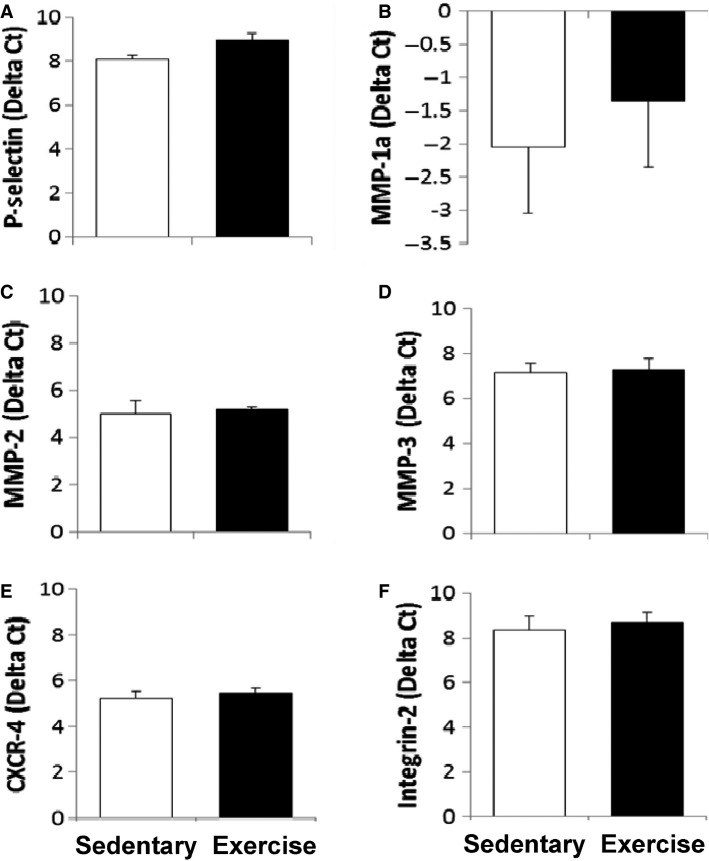
Acute Aerobic Exercise Does Not Alter Cell Adhesion Molecules. There was no significant difference in any marker of cell adhesion molecules between exercise and sedentary mice. MMP: matrix metalloproteinase, CXCR-4: chemokine receptor type 4.

We tested whether the increased BMC retention with exercise was related to expression of some key cell adhesion molecules. As illustrated in Figure3, transcription abundance of P-selectin, MMP-1a, MMP-2, MMP3, CXCR-4, and integrin-2 were similar between groups.

### Chronic exercise training

#### Physical characteristics, cardiac function, and exercise capacity

**Figure 4:**
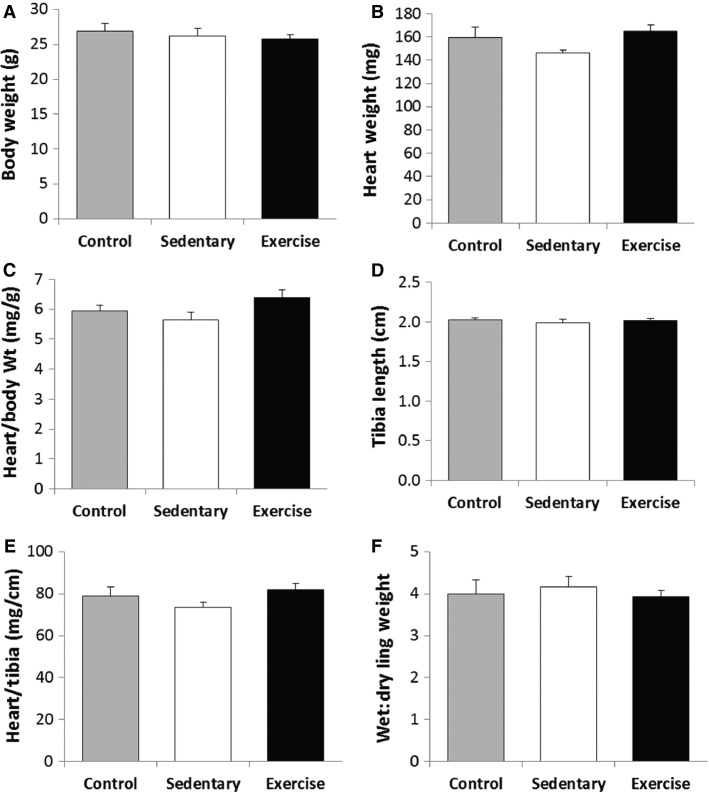
Chronic Exercise Training and Physical Characteristics. The physical characteristics of all groups were similar following chronic exercise training.

**Figure 5:**
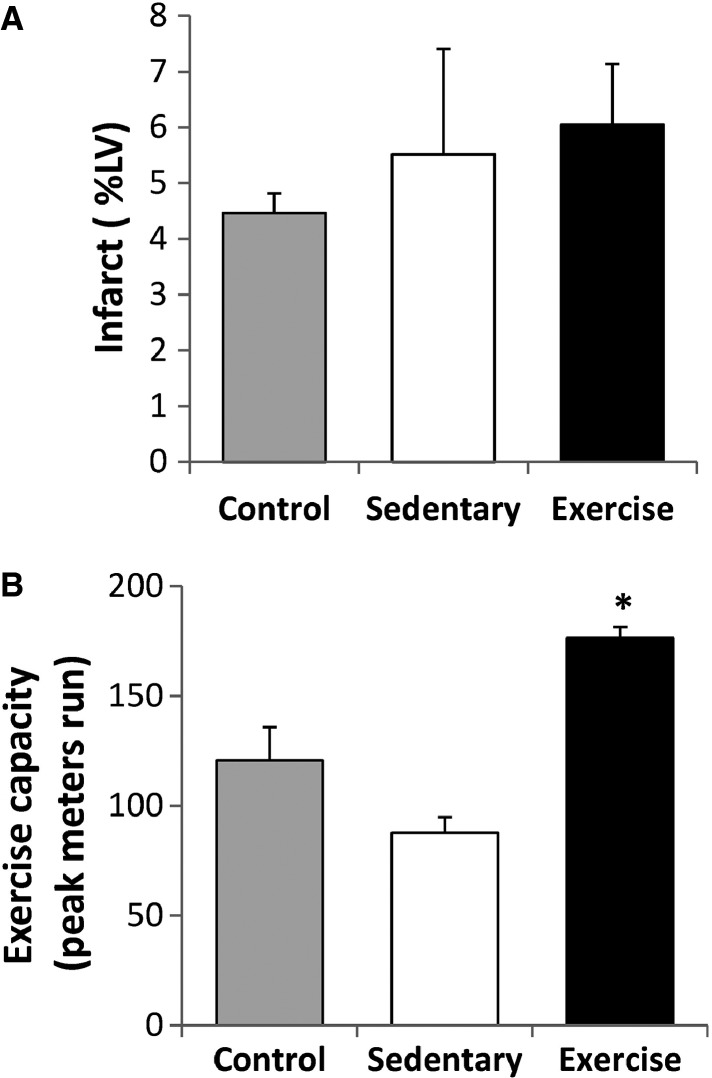
The Effects of Chronic Exercise Training on Infarct Size and Exercise Tolerance. Infarct size was similar among groups (Panel A). Exercise training increased exercise capacity relative to sedentary animals (Panel B). * indicates *P* < 0.05 versus sedentary.

As shown in Figure4, there were no differences for physical characteristics across experimental groups. Compared to sedentary animals, chronic exercise training increased exercise capacity (*P* < 0.05) but it did not alter LVEF, infarct size, or regional wall thickness (*P *= NS) (Fig.5 and Table3). While BMC’s alone did not affect exercise capacity, they did increase LVEF (*P* < 0.05) relative to vehicle controls (Table3).

**Table 3:** Post training echocardiography

	Control (*n* = 4)	Sedentary (*n* = 6)	Exercise (*n* = 7)	*P*-value
IVS;d (mm)	1.11 ± 0.1	1.00 ± 0.0	1.09 ± 0.1	NS
LVID;d (mm)	3.65 ± 0.4	3.73 ± 0.02	3.49 ± 0.2	NS
LVPW;d (mm)	1.02 ± 0.1	1.05 ± 0.1	1.15 ± 0.1	NS
IVS;s (mm)	1.44 ± 0.2	1.43 ± 0.1	1.45 ± 0.2	NS
LVID;s (mm)	2.81 ± 0.3	2.58 ± 0.3	2.41 ± 0.03	NS
LVPW;s (mm)	1.39 ± 0.1	1.38 ± 0.2	1.49 ± 0.2	NS
LV Vol;d (*μ*L)	58.7 ± 12	61.3 ± 9.3	52.4 ± 7.1	NS
LV Vol;s (*μ*L)	31.2 ± 7.2	26.9 ± 7.9	22.7 ± 5.0	NS
Stroke volume (*μ*L)	27.5 ± 6.0	34.3 ± 2.3	29.7 ± 2.6	NS
EF (%)	46.2 ± 4.8	60.0 ± 5.9	60.2 ± 5.2	0.05
LV Mass (mg)	152 ± 16	155 ± 25	152.3 ± 9.9	NS
MV E	264 ± 25	228 ± 51	287 ± 51	NS
MV A	183 ± 18	193 ± 46	198 ± 17	NS
MV E/A	1.48 ± 0.2	1.17 ± 0.1	1.43 ± 0.2	NS

IVS, interventricular septum; LVID, left ventricular internal diameter; LVPW, left ventricular posterior wall; d, diastole; s, systole; EF, ejection fraction; MV, mitral valve; E/A, early to late ventricular filling.

#### Ki67^+^ nuclei

**Figure 6:**
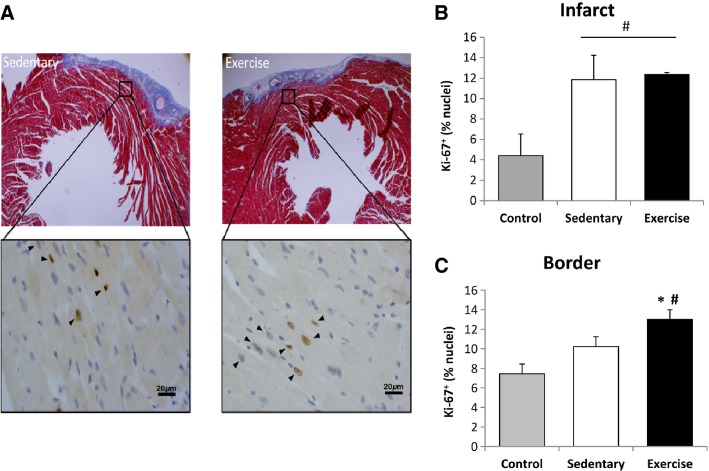
Chronic Exercise Training and BMC Injections Increase Ki67^+^ Cell Abundance. Counts of Ki-67^+^, a marker of proliferation, were increased in the infarct zone compared to control mice (mice not receiving cell infusions). Exercise training increased Ki-67^+^ counts in the border zone compared to sedentary and control mice. * indicates *P* < 0.05 versus sedentary, # indicates *P* < 0.05 versus control.

Ki67^+^ nuclei were counted in the infarct and border zone regions of the heart. Injection of BMC’s increased Ki67^+^ cells in the infarct region (Fig.6, Panel B) and the border zones of the heart (*P* < 0.05), an effect that was potentiated with chronic exercise training (*P* < 0.05) Figure6, Panel C).

## Discussion

Cell therapy for heart disease holds great therapeutic promise (Beltrami et al. 2001; Orlic et al. 2003; Dimmeler et al. 2008; Ott et al. 2008; Passier et al. 2008; Dib et al. 2010; Hatzistergos et al. 2010; Chugh et al. 2012). However, one of the limiting factors in maximizing the efficacy of cell therapy is having a high percentage of the infused cells home, engraft, and differentiate in the heart (Chavakis et al. 2008). In this study, we demonstrated that BMC infusion augments LVEF versus noninfused vehicle controls. We also observed that BMC retention was enhanced if BMC infusions were coupled with acute aerobic exercise. In a mouse model of small epicardial MI, our results show that acute exercise increases in vivo myocardial BMC retention in the infarct zone of the heart following intravenous infusions; with some of the retained BMC’s showing co-expression with alpha sarcomeric actin. This was not associated with shifts in the abundance of key cell adhesion molecules. We also show that BMC infusions alone did not alter cardiac remodeling or exercise capacity 5 weeks post infusion, despite enhancing LV ejection fraction. Exercise capacity was enhanced following training, and exercise increased the number of Ki67^+^ nuclei in the border zone, but not the infarcted zone of the heart. Our findings support the original hypothesis that aerobic exercise increases myocardial retention of exogenously infused GFP^+^ bone marrow-derived cells in the heart and may stimulate cardiomyocytes to enter the cell cycle.

Stem cell therapy has been well documented to favorably impact functional capacity, LV function, and remodeling of the infarcted heart (Cosmo et al. 2012; Duran et al. 2013; Sanganalmath and Bolli 2013). In our study, BMC administration alone did not alter exercise capacity, regional wall thickness, infarct size, or LV mass 5 weeks after infusion. This may be due to the relatively low cell infusion number, the small cryoinjury MI’s induced in our model, or the short period of time for follow-up. It is interesting to note that despite these issues, BMC’s did, however, lead to an increase in LV ejection fraction and Ki67^+^ nuclei number in the infarcted and border zone of the heart.

In our small nontransmural MI model, GFP^+^ BMC retention was enhanced with exercise in the infarcted zone of the LV, but not in the border or distant zones. This finding is consistent with cell homing toward the site of myocardial injury (Elser et al. 2012). In fact, the overall low BMC retention in the noninfarcted regions of the heart often elicited undetectable PCR quantification of GFP^+^ and SRY genes. One plausible scenario is that BMC’s originally infiltrated viable myocardium before migrating to the site of injury, resulting in a low number of BMC’s in the remote regions of the heart. Nonetheless, the augmented early BMC retention with acute exercise did not alter cardiac function at baseline 24 h post BMC infusion.

Acute exercise has been shown to induce a host of systemic mechanical, metabolic, neurohumoral, and paracrine responses. Acute aerobic exercise increases heart rate, stroke volume, blood pressure, cardiac output, and coronary flow via enhanced sympathetic outflow (Bevegard et al. 1963). Acute exercise also increases substrate flux, potentiates inflammation and oxidative stress, and induces an array of endocrine responses. Thus, acute exercise invokes a wide array of potential mediators, including alterations in cytokines and cell adhesion molecules, that may promote progenitor cell retention and cardiomyocyte proliferation in the heart (Bevegard et al. 1963; Peled et al. 1999; Okutsu et al. 2005; Sandri et al. 2005; Boström et al. 2010; Erbs et al. 2010).

Bone marrow cells are mobilized in vivo from bone marrow for tissue repair following injury. Acute exercise may have increased BMC mobilization by increasing oxidative stress and inflammation thus triggering repair mechanisms (Sandri et al. 2005; Erbs et al. 2010). BMC mobilization, particularly in response to tissue injury occurs in several stages, with an initial progenitor release from the bone marrow niche and subsequent migration across the sinusoidal endothelium. The bone marrow retention of hematopoietic stem cells is critically regulated by stromal cell-derived factor 1*α* (SDF-1) and its receptor CXCR4 (Peled et al. 1999). Studies have shown an upregulation of serum SDF-1/CXCR4 with acute and chronic exercise in healthy volunteers (Okutsu et al. 2005), heart failure patients (Sandri et al. 2005; Erbs et al. 2010), and post-MI patients (Brehm et al. 2009), suggesting a role for the SDF-1/CXCR4 axis in exercise-induced BMC retention. Though we did not observe increased abundance of the transcript encoding cell adhesion molecules, we cannot exclude post-transcriptional effects on adhesion molecules and their interactions with injected and/or mobilized BMC’s.

From a probability perspective, the several-fold increase in cardiac output and coronary flow during and after acute exercise augments the overall exposure of infused progenitors to the heart. Moreover, the redistribution of cardiac output to the heart via exercise-induced splanchnic vasoconstriction may also enhance the probability of progenitor cell exposure for retention in the heart. However, in our studies in which cardiac output was increased in sedentary animals using dobutamine, followed by the infusion of 5 × 10^6^ bone marrow cells, we were unable to achieve the enhanced BMC engraftment observed with exercise prior to BMC infusion. This suggests that factors beyond coronary flow are perhaps involved in the enhanced BMC retention with exercise. Further studies are needed to identify the associated mechanisms.

To our knowledge, only one small study to date has looked at the impact of chronic exercise training on stem cell therapy after MI (Cosmo et al. 2012). Cosmo et al. (2012) found that 30 days of swimming post-MI increased LVEF and improved adverse ventricular remodeling following mononuclear cell transplantation in the heart. It is known, however, that swimming elicits different cardiovascular responses than other forms of aerobic exercise and hence may not be translationally applicable to patients (Flaim et al. 1979). In the present study, the finding that chronic treadmill running increased the number of Ki67^+^ cells is encouraging, but these findings did not lead to improved LV function or remodeling. Examining how different exercise modalities impact the efficacy of cell therapy will be an important undertaking for future studies.

There are several limitations to our experiments. First we did not include a vehicle control group in the acute aerobic exercise studies, hence limiting our ability to ascertain the acute effects of BMC’s on the heart. Second, the induced cryoinjury MI was very small in our model, and did not result in overt functional impairments. We acknowledge that both serial measures of cardiac function over time and the inclusion of a non-MI sham group would have improved our sensitivity in tracking cardiac function postcryoinjury. Longer term training studies in larger infarct models are warranted to test whether training can improve cardiomyocyte regeneration enough to positively influence LV function. Another limitation to our study is that we infused a relatively small number of BMC’s and assessed the functional phenotype after a only brief time interval. Future studies could better test the efficacy of exercise and cell therapy by extending the number of infused BMCs and the duration of follow-up to quantify retention. A greater number of infused cells would also allow for cell retention to be quantified with techniques beyond immunohistochemistry, that is, SRY gene abundance. This is an important limitation of this study, as autofluorescence limited our ability to adequately examine cell retention following the chronic aerobic exercise training arm of the study. Moreover, exclusively relying on immunohistochemistry methodology makes it impossible to accurately assess GFP^+^ BMC retention in the whole heart, as only a few representative cross sections can be analyzed. A positive attribute of our study is that we used middle age mice, that is, 12 months of age, to mimic the clinical situation by which cell therapy and exercise might most benefit patients. Different outcomes might occur in younger or older mice, and should be tested in future work.

In summary, acute aerobic exercise increases the retention of exogenously infused BMC’s in mouse myocardium following MI. Animals treated with chronic exercise training exhibited increased exercise capacity and cardiac mass without any change in LVEF, infarct size, or regional wall thickness relative to sedentary controls. While BMC’s alone did not improve exercise capacity, they did increase LVEF and Ki67^+^ nuclei number in the border zone of the heart, an effect that was increased with chronic exercise training. We conclude that acute aerobic exercise increases BMC retention in infarcted hearts and that chronic training increases exogenous BMC-mediated effects on stimulating the cardiomyocyte cell cycle.

### Translational significance

These preclinical results suggest that exercise may be an efficacious adjunct to optimize myocardial cell mobilization and/or retention in cell therapy. Further work is required to test the long term impact of exercise training upon BMC retention and functional consequences of exercise in cell therapy, particularly on cardiomyocyte proliferation in larger, more severe infarcts.

## References

[b1] Barbash IM, Chouraqui P, Baron J, Feinberg MS, Etzion S, Tessone A (2003). Systemic delivery of bone marrow-derived mesenchymal stem cells to the infarcted myocardium: feasibility, cell migration, and body distribution. Circulation.

[b2] Beltrami AP, Urbanek K, Kajstura J, Yan SM, Finato N, Bussani R (2001). Evidence that human cardiac myocytes divide after myocardial infarction. N. Engl. J. Med.

[b3] Bevegard S, Holmgren A, Jonsson B (1963). Circulatory studies in well trained athletes at rest and during heavy exercise. With special reference to stroke volume and the influence of body position. Acta Physiol. Scand.

[b4] Boström P, Mann N, Wu J, Quintero PA, Plovie ER, Panáková D (2010). C/EBP*β* controls exercise-induced cardiac growth and protects against pathological cardiac remodeling. Cell.

[b5] Brehm M, Picard F, Ebner P, Turan G, Bölke E, Köstering M (2009). Effects of exercise training on mobilization and functional activity of blood-derived progenitor cells in patients with acute myocardial infarction. Eur. J. Med. Res.

[b6] Chavakis E, Urbich C, Dimmeler S (2008). Homing and engraftment of progenitor cells: a prerequisite for cell therapy. J. Mol. Cell. Cardiol.

[b7] Chugh AR, Beache GM, Loughran JH, Mewton N, Elmore JB, Kajstura J (2012). Administration of cardiac stem cells in patients with ischemic cardiomyopathy: the SCIPIO trial: surgical aspects and interim analysis of myocardial function and viability by magnetic resonance. Circulation.

[b8] Cosmo S, Francisco JC, da Cunha RC, de Macedo RM, Faria-Neto JR, Simeoni R (2012). Effect of exercise associated with stem cell transplantation on ventricular function in rats after acute myocardial infarction. Rev. Bras. Cir. Cardiovasc.

[b9] Dib N, Menasche P, Bartunek JJ, Zeiher AM, Terzic A, Chronos NA, International Society for Cardiovascular Translational Research (2010). Recommendations for successful training on methods of delivery of biologics for cardiac regeneration: a report of the International Society for Cardiovascular Translational Research. JACC Cardiovasc. Interv.

[b10] Dimmeler S, Burchfield J, Zeiher AM (2008). Cell-based therapy of myocardial infarction. Arterioscler. Thromb. Vasc. Biol.

[b11] Duran JM, Makarewich CA, Sharp TE, Starosta T, Zhu F, Hoffman NE (2013). Bone-derived stem cells repair the heart after myocardial infarction through transdifferentiation and paracrine signaling mechanisms. Circ. Res.

[b12] Elser JA, Purcell BP, Allana IA, Burdick JA, Margulies KB (2012). Ischemia induces P-selectin-mediated selective progenitor cell engraftment in the isolated-perfused heart. J. Mol. Cell. Cardiol.

[b13] Erbs S, Höllriegel R, Linke A, Beck EB, Adams V, Gielen S (2010). Exercise training in patients with advanced chronic heart failure (NYHA IIIb) promotes restoration of peripheral vasomotor function, induction of endogenous regeneration, and improvement of left ventricular function. Circ. Heart Fail.

[b14] Flaim SF, Minteer WJ, Clark DP, Zelis R (1979). Cardiovascular response to acute aquatic and treadmill exercise in the untrained rat. J. Appl. Physiol. Respir. Environ. Exerc. Physiol.

[b15] Hatzistergos KE, Quevedo H, Oskouei BN, Hu Q, Feigenbaum GS, Margitich IS (2010). Bone marrow mesenchymal stem cells stimulate cardiac stem cell proliferation and differentiation. Circ. Res.

[b16] Kolwicz SC, MacDonnell SM, Renna BF, Reger PO, Seqqat R, Rafiq K (2009). Left ventricular remodeling with exercise in hypertension. Am. J. Physiol. Heart Circ. Physiol.

[b17] Kwak H-B, Song W, Lawler JM (2006). Exercise training attenuates age-induced elevation in Bax/Bcl-2 ratio, apoptosis, and remodeling in the rat heart. FASEB J.

[b18] Mishra PK (2008). Bone marrow-derived mesenchymal stem cells for treatment of heart failure: is it all paracrine actions and immunomodulation?. J. Cardiovasc. Med. (Hagerstown).

[b19] Nygren JM, Jovinge S, Breitbach M, Säwén P, Röll W, Hescheler J (2004). Bone marrow-derived hematopoietic cells generate cardiomyocytes at a low frequency through cell fusion, but not transdifferentiation. Nat. Med.

[b20] Oh H, Bradfute SB, Gallardo TD, Nakamura T, Gaussin V, Mishina Y (2003). Cardiac progenitor cells from adult myocardium: homing, differentiation, and fusion after infarction. Proc. Natl Acad. Sci. U. S. A.

[b21] Okutsu M, Ishii K, Niu KJ, Nagatomi R (2005). Cortisol-induced CXCR4 augmentation mobilizes T lymphocytes after acute physical stress. Am. J. Physiol. Regul. Integr. Comp. Physiol.

[b22] Orlic D, Kajstura J, Chimenti S, Bodine DM, Leri A, Anversa P (2003). Bone marrow stem cells regenerate infarcted myocardium. Pediatr. Transplant.

[b23] Ott HC, Matthiesen TS, Goh S-K, Black LD, Kren SM, Netoff TI (2008). Perfusion-decellularized matrix: using nature’s platform to engineer a bioartificial heart. Nat. Med.

[b24] Passier R, van Laake LW, Mummery CL (2008). Stem-cell-based therapy and lessons from the heart. Nature.

[b25] Peled A, Petit I, Kollet O, Magid M, Ponomaryov T, Byk T (1999). Dependence of human stem cell engraftment and repopulation of NOD/SCID mice on CXCR4. Science.

[b26] Rehman J, Li J, Parvathaneni L, Karlsson G, Panchal VR, Temm CJ (2004). Exercise acutely increases circulating endothelial progenitor cells and monocyte-/macrophage-derived angiogenic cells. J. Am. Coll. Cardiol.

[b27] Sandri M, Adams V, Gielen S, Linke A, Lenk K, Kränkel N (2005). Effects of exercise and ischemia on mobilization and functional activation of blood-derived progenitor cells in patients with ischemic syndromes: results of 3 randomized studies. Circulation.

[b28] Sanganalmath SK, Bolli R (2013). Cell therapy for heart failure: a comprehensive overview of experimental and clinical studies, current challenges, and future directions. Circ. Res.

[b29] Segers VFM, Lee RT (2008). Stem-cell therapy for cardiac disease. Nature.

[b30] Sturgeon K, Muthukumaran G, Ding D, Bajulaiye A, Ferrari V, Libonati JR (2015). Moderate intensity treadmill exercise training decreases murine cardiomyocyte cross sectional area. Physiol. Rep.

